# Quantifying phenology and migratory behaviours of hummingbirds using single-site dynamics and mark-detection analyses

**DOI:** 10.1098/rspb.2022.0991

**Published:** 2022-09-14

**Authors:** Simon G. English, Scott Wilson, Ruta R. Bandivadekar, Emily E. Graves, Marcel Holyoak, Jennifer C. Brown, Lisa A. Tell

**Affiliations:** ^1^ Department of Veterinary Medicine and Epidemiology, University of California Davis, 1 Shields Avenue, Davis, CA 95616, USA; ^2^ Department of Environmental Science and Policy, University of California Davis, Davis, CA, USA; ^3^ Department of Forest and Conservation Sciences, University of British Columbia, Vancouver, British Columbia, Canada; ^4^ Wildlife Research Division, Environment and Climate Change Canada, Delta, British Columbia, Canada; ^5^ United States Fish and Wildlife Service, Sacramento, CA, USA

**Keywords:** multi-state open robust design with state uncertainty, partial migration, Anna’s hummingbirds (*Calypte anna*), Allen’s hummingbirds (*Selasphorus sasin*), radio-frequency identification, facultative migration

## Abstract

Nuanced understanding of seasonal movements of partially migratory birds is paramount to species and habitat conservation. Using nascent statistical methods, we identified migratory strategies of birds outfitted with radio-frequency identification (RFID) tags detected at RFID feeders in two sites in California, USA. We quantified proportions of migrants and residents and the seasonal phenology for each movement strategy in Allen’s and Anna’s hummingbirds; we also validated our methodology by fitting our model to obligate migratory black-chinned hummingbirds. Allen’s and Anna’s hummingbirds exhibited characteristics of facultative migratory behaviour. We also quantified apparent annual survival for each migratory strategy and found that residents had significantly higher probabilities of apparent survival. Low survival estimates for migrants suggest that a high proportion of birds in the migrant group permanently emigrated from our study sites. Considered together, our analyses suggest that hummingbirds in both northern and southern California sites partake in diverse and highly plastic migratory behaviours. Our assessment elucidates the dynamics underlying idiosyncratic migratory behaviours of two species of hummingbirds, in addition to describing a framework for similar assessments of migratory behaviours using the multi-state open robust design with state uncertainty model and single-site dynamics.

## 1. Background

Birds are the most mobile of all animal taxa, and migratory behaviour is one of the most extensively studied phenomena in ornithology [1]. Despite persistent international effort to quantify the idiosyncratic patterns of seasonal movements of birds [2], there are still areas of migratory behaviour that are remarkably poorly understood. Fascination with bird migration relates to an urgent need to understand movements throughout the annual cycle as well as the drivers of migration in light of major population transformations [3–5] and climate change related effects on the life cycle of birds [6–8]. When breeding habitat is not rich enough to support birds over their full annual cycle, birds gain a fitness advantage by migrating [9]; however, migratory birds are especially vulnerable to effects of climate change and habitat disturbance [10,11], partly due to their reliance on high quality stopover sites [12], overwintering habitat [13], and the perils of migration [14,15]. Conversely, birds may gain a fitness advantage by adopting a resident strategy [16] but in exchange are exposed to threats including high mortality rates from extreme weather events and potentially restricted resource availability [9,17].

Residency and complete migration represent two extremes along a spectrum of migration strategies employed by birds [18]. It is increasingly apparent that diverse migratory strategies are commonly adopted within species of avifauna, called partial migration [19]. Moreover, various seasonal movement strategies may be employed within the life cycle of adaptive individuals [18,20]. The ability of some individuals to adaptively employ alternative migratory strategies as resources and environmental conditions demand, called facultative migration [9,16], may grant individuals a fitness advantage under variable environmental conditions [21].

Hummingbirds (*Trochilidae*) in North America exhibit a high degree of diversity and still are rapidly diversifying [22]. As such, hummingbirds exploit diverse ecological niches, where divergent migratory strategies are undertaken among species utilizing the same breeding habitat. For instance, three common hummingbirds in California USA, Anna’s hummingbirds (*Calypte anna*), black-chinned hummingbirds (*Archilochus alexandri*) and Allen’s hummingbirds (*Selasphorus sasin*) can all be reliably encountered within the same summer breeding area [23]; however, each partakes in different migratory strategies. Anna’s hummingbirds are known to engage in migratory movement after the breeding season, although the extent of this movement varies among individuals and populations [24]. Movement patterns of Anna’s hummingbird include post-breeding altitudinal migration to high elevations and southeastward migrations from California to Arizona and New Mexico [24]. Black-chinned hummingbirds are predominantly medium-distance migrants, migrating through the southeastern Sierra Nevada mountains to Mexico from California, USA [25]. Mainland populations of Allen’s hummingbirds are short- to medium-distance migrants where breeding occurs on the Pacific coast of California after which individuals move to wintering grounds in Northern and Central Mexico (*S. s. sasin*) [26]. Allen’s hummingbirds are unusual since the purported subspecies that exists primarily in California’s Channel Islands does not migrate (*S. s. sedentarius*) [27]. For each of these species, while multiple migratory strategies are acknowledged in the literature [3,19,24,26–28], all accounts of migratory behaviours are of a qualitative nature.

Migratory demography and phenology are notoriously difficult to quantify, particularly in study areas with co-inhabitant populations of migratory and resident birds, and birds may adopt either strategy from one year to the next [29]. Novel methods for analysing mark–recapture data have recently been extended to rigorously account for alternative movement strategies by including state-uncertainty in multistate models [30–32]. This class of models is useful for the quantification of population dynamics among species with diverse observable and unobservable discrete states, including migratory, breeding, and age states, where individual animals may transition among states between years [33]. These methods can be further extended to account for any number of distinct migratory behaviours, thus allowing for highly nuanced assessments of migratory plasticity within complex migrant systems.

We used a novel multi-state model with state uncertainty to examine migratory strategies of coexisting Anna’s and Allen’s hummingbirds, and compared with obligate migratory black-chinned hummingbirds. Because the models probabilistically assign individuals to unobservable states, these multi-state models are powerful tools for quantifying population structures among animals adopting diverse seasonal movement strategies [31,34]. We analysed data from two sites, in northern and southern California, USA, to determine if proportional composition of migrants and residents was species- or population-specific. We additionally quantified the probability of adopting alternative movement strategies in consecutive years, as in facultative migration and the probabilities of survival associated with each movement strategy. Finally, we estimated phenological parameters of migratory birds that relate to the seasonal movement patterns of individuals, including arrival, departure and residency times.

## 2. Methods

### (a) Study sites

Hummingbirds were captured using feeder Hall traps [35] and tagged with radio-frequency identification (RFID) tags at two sites in California, USA. Our southern site, located in Beverley Hills (BH) at $34.10^{\circ }\;N$ and $118.41^{\circ }\;W$, had six hummingbird feeder stations outfitted with RFID readers, and data were collected from 1 October 2017 to 1 October 2021 (electronic supplementary material, figure S1). Our northern site, located in Winters (SB) at $38.53^{\circ }\;N$ and $121.85^{\circ }\;W$, had three hummingbird feeder stations outfitted with RFID readers, and data were collected from 1 September 2016 to 1 November 2021. Detailed information on the use of RFID technology with hummingbird feeder receiver stations are described by Bandivadekar *et al.* [36]. We analysed a subset of data collected from both sites spanning 1 February 2018 to 31 January 2021 such that annual and monthly detection data at both sites could be divided among complete primary (annual) and secondary (monthly) sampling occasions (electronic supplementary material, figure S1).

Tagging at BH took place on an average of 1.8 d mo^−1^ ± 0.6 d mo^−1^ during the autumn banding season (August–October), 0.3 d mo^−1^ ± 0.3 d mo^−1^ in the winter season (November–January), 0.7 d mo^−1^ ± 0.6 d mo^−1^ in the spring (February–April) and 0.4 d mo^−1^ ± 0.6 d mo^−1^ in the summer (May–July). The tagging effort resulted in an average of 41 ± 15 new birds tagged per month in the autumn, 10 ± 10 birds per month in the winter, 5 ± 4 birds per month in the spring, and 4 ± 4 birds per month in the summer across the 3 years. At SB, tagging took place on an average of 1.8 d mo^−1^ ± 0.4 d mo^−1^ in the autumn, 0.7 d mo^−1^ ± 0.6 d mo^−1^ in the winter, 1.8 d mo^−1^ ± 0.7 d mo^−1^ in the spring, and 3.1 d mo^−1^ ± 0.6 d mo^−1^ in the summer. The tagging effort at SB resulted in an average of 10 ± 4 birds per month in the autumn, 3 ± 1 birds per month in the winter, 5 ± 2 birds per month in the spring and 25 ± 8 birds per month in the summer across both years. RFID tagging at both sites occurred between 06.00 and 12.00 hours by one to two trained individuals.

### (b) Study species

We analysed capture-detection data for 540 Anna’s, 330 Allen’s and 177 black-chinned hummingbirds across both study sites between 1 February 2018 and 31 January 2021. Of these data, 19 Anna’s and 7 Allen’s hummingbirds were first tagged before the beginning of the study period. Allen’s hummingbirds were detected in our southern California site as well as 318 Anna’s hummingbirds. At our northern California site, we collected data from 222 Anna’s hummingbirds and all 177 black-chinned hummingbirds (our northern site was outside of the range of Allen’s hummingbirds). Each site was considered as a separate hummingbird population because neither site ever detected a hummingbird tagged at the other site. Tags were implanted subcutaneously following protocols for passive integrated transponder applications in hummingbirds [36].

### (c) Statistical methods

To estimate the proportional composition of each species in the two populations, we fit a multi-state open robust design with state-uncertainty (MSORD-SU) model [31]. Open robust design models comprise primary periods (years) during which the population is assumed open, and secondary periods (months) during which the population is assumed closed. Statistical modelling was executed using program MARK [37] interfaced from the R [38] package RMark v. 2.2.7 [39]. The MSORD-SU model had two possible states: winter residents and year-round residents (hereafter ‘residents’; M) and breeding migrants (hereafter ‘migrants’; M). Residents were individuals that were present in our study area between the months of November and January because they did not migrate and therefore were present in the study region year-round or else entered the study area following post-breeding movements from outside the study area. These may have included individuals from more northern breeding sites, altitudinal migrants or individuals that bred nearby but outside of the broader study area. Residents therefore also have entry probabilities in our study sites. Migrants were those individuals that were assumed not to be present in the winter months (November–January). We configured the annual period to begin in February and end in January to estimate seasonal movements over contiguous, biologically meaningful seasons. The annual period of the MSORD-SU model therefore ends after all migratory black-chinned hummingbirds departed from the study area and before these obligate migrants return. Although the phenology of each species at our study sites is unique, the selected winter timing coincided with an observed decline in abundance in Allen’s and Anna’s hummingbirds at our sites. Therefore, we defined any individual detected in the winter months as a known resident (*δ* = 1), while birds detected during the other periods (February–October) were assigned an unknown state because either residents or migrants could be present during those months (*δ* = 0).

We tested covariates of species, site and seasonal movement strategy as well as the expected time since arrival (TSA) on the study area and a quadratically transformed term for the monthly interval (quad) in candidate parametrizations of *β* and Φ (electronic supplementary material, table S1). The TSA term was computed as the subtraction of the monthly entry cohort from the monthly interval [32]. This model separately estimates (1) apparent annual survival (*S*) from year *t* to year *t* + 1 (representing site fidelity and true survival), (2) the probability of transitioning between states ($\Psi^{R+1}$) in years *t* and *t* + 1, (3) the proportional composition of states (*ω*^*R*^) within populations in year *t*, (4) the probability of persistence in the study area ($\Phi^{j}$), representing the probability of being in the study area in secondary period *j*, given that it was present in secondary period *j* − 1, and (5) the monthly probability of entry (*β*), representing the probability of entering the study area in secondary period *j*. This model also includes parameters to characterize observation processes including (6) the probability of correctly classifying the state of an individual, given that it is detected (*δ*), (7) the probability of being in state *R*, given that the state upon detection in year *t* was unknown (*π*^*R*^) and (8) the monthly probability of detection (*ρ*^*j*^).

The MSORD-SU model further derives five additional parameters from the parameters estimated directly from data: (1) superpopulation size (*N*; defined as the number of individuals using the site for one or more months in primary period *t*), (2) intensity of availability (*α*; defined as the probability that an individual in year *t* is present and available for detection in month *j*) [40], (3) two methods to estimate residency time (*R*_1_ and *R*_2_), (4) expected arrival time (*A*), and (5) expected departure time (*D*). Superpopulation size is estimated as a function of the number of tagged individuals of each species and state corrected for imperfect detection (*ρ*). However, because capture probabilities were not constant or uniform and represent a separate process from the estimation of detection probabilities, *ρ*, we do not report superpopulation size. To estimate residency time, *R*_1_ is derived using estimates of Φ and *β*, and assumes that arrival and departure from the study site is bounded by sampling effort [32]. Our analyses satisfied this assumption because our use of the RFID tags meant that birds were remotely detected year-round via the RFID feeders. The second method, *R*_2_ is derived using only Φ estimates, and assumes a negligible probability that an individual will remain on the study area for the full primary period. Both methods for the derivation of residency time are presented by Kendall *et al.* [32]. Probabilities of detecting birds on tagging days were assumed equal to detection-only days, since all available detectors were active regardless of tagging effort.

Since the MSORD-SU model and similar models estimate many parameters simultaneously, these tools can be prohibitively data-hungry [31,32,41]. To accommodate this restrictive property of the MSORD-SU model, we applied biologically meaningful parameter constraints, thus reducing the parameter space [31,42]. To apply this structure to migratory and resident birds within our study area, we assumed resident birds persisted on the study area with a probability of 1, from the time of entry, through winter months to the end of the annual primary period (November–February; $\Phi^{R}=1$). Implicit in this assumption is that residents comprise multiple possible seasonal movement strategies, including short- and long-distance post-breeding dispersing birds that arrive on our study area, and annually stationary resident birds. We are currently unable to differentiate between these strategies with this modelling approach. Conversely, the migrant class birds were allowed to depart from the study area in any monthly interval preceding the end of the annual period. Program MARK does not allow users to assign different annual periods to different individuals within the same MSORD-SU model; we therefore were unable to assign species-specific breeding and non-breeding stages throughout the annual period.

There are currently no comprehensive goodness-of-fit tests available for this model, and thus we were unable to evaluate the overall fit of the MSORD-SU model to our data [31,32,41], and instead selected the top model based on the AICc from a series of candidate models [43]. We selected the top model based on the subset of data excluding black-chinned hummingbirds because black-chinned hummingbirds were expected to yield boundary or singular estimates for the resident class for all parameters (electronic supplementary material, table S1). Using this top model, we then reanalysed the full dataset including black-chinned hummingbird data to validate our methodology by confirming that all black-chinned hummingbirds were correctly classified as migrants. Raw log-odds and multinomial logit scale *B* estimates were considered significantly different from the intercept when 95% confidence intervals (CI) did not overlap 0. Standard errors for complementary proportions of *ω* estimates were derived using the delta method [44]. Estimates are presented as the mean ± s.e., unless otherwise indicated.

## 3. Results

### (a) Hummingbird population structure

**Figure 1.**
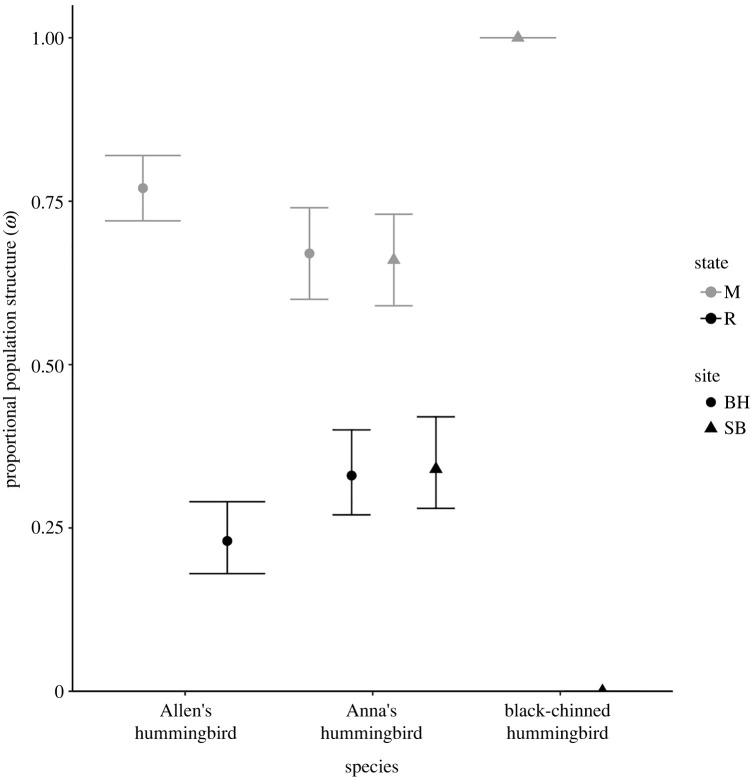
Proportion of migratory (M) and resident (R) individuals in populations of Anna’s (*C. anna*), Allen’s (*S. sasin*) and black-chinned (*A. alexandri*) hummingbirds for our northern (SB) and southern (BH) study sites in California, USA. Vertical error bars on point estimates are the upper and lower bounds of 95% confidence intervals.

Proportions of residents in populations of Anna’s hummingbirds were not significantly different compared to Allen’s hummingbirds ($\omega_{\mathrm{Ann}a^{\prime }s}^{B}=0.3\pm 0.2$; CI : −0.09  to  0.76), or in the proportion of resident Anna’s hummingbirds at our northern site compared to our southern site ($\omega_{\mathrm{Site}}^{B}=-0.3\pm 0.2$; CI : −0.75  to  0.22). We estimated that 0.19 ± 0.02 (CI: 0.15–0.25) of the Allen’s hummingbird population in our Southern California site were residents and thus, 0.81 ± 0.02 (CI: 0.76–0.85) were estimated to be migratory. Effectively all black-chinned hummingbirds were classified as migrants, as the proportion of residents was estimated at the lower boundary on the multinomial logit scale ($\omega_{\mathrm{black}\text{-}\mathrm{chin}}^{B}=-18\pm 2163$; CI: −4258  to  4222) and could not be precisely estimated (figure 1).

At our southern site, 0.25 ± 0.03 (CI: 0.20–0.32) of Anna’s hummingbirds were resident and 0.75 ± 0.03 (CI: 0.69–0.81) were migratory. At our northern site, 0.21 ± 0.03 (CI: 0.15–0.27) of Anna’s hummingbirds were resident and 0.79 ± 0.03 (CI: 0.73–0.86) were migratory.

### (b) Annual probability of state transition

Probabilities of changing seasonal movement strategies between annual periods included a term for species, where Anna’s hummingbirds had significantly lower probabilities of transitioning than Allen’s hummingbirds ($\Psi_{\mathrm{Ann}a^{\prime }s}^{B}=-0.66\pm 0.25$; CI : −1.2  to  −0.17). Site did not significantly predict transition probability for Anna’s hummingbirds ($\Psi_{\mathrm{site}}^{B}=-0.27\pm 0.31$; CI : −0.87  to  0.33). Allen’s hummingbirds had a probability of changing seasonal movement strategies of 0.58 ± 0.04 (CI: 0.49–0.67). Anna’s hummingbirds had transition probabilities of 0.42 ± 0.04 (CI: 0.34–0.50) at our southern site and 0.35 ± 0.06 (CI: 0.25–0.47) at our northern site.

### (c) Apparent annual probability of survival

Hummingbirds in the resident class had significantly higher probabilities of apparent annual survival compared with those in the migrant class ($S_{R}^{B}=3.8\pm 0.4$; CI: 3.1–4.5). Apparent survival was also significantly higher in the northern site compared to the southern site ($S_{\mathrm{site}}^{B}=1.5\pm 0.4$; CI: 0.77–2.3); however, species did not significantly predict annual survival ($S_{\mathrm{Ann}a^{\prime }s}^{B}=-0.3\pm 0.2$; CI: –0.80 to 0.11). Probability of Allen’s hummingbird apparent annual survival was estimated at 0.73 ± 0.04 (CI: 0.66–0.80) for residents and 0.06 ± 0.02 (CI: 0.03–0.11) for migrants. For Anna’s hummingbirds in southern California, apparent annual survival was estimated at 0.66 ± 0.03 (CI: 0.59–0.72) for residents and 0.04 ± 0.02 (CI: 0.02–0.08) for migrants. Anna’s hummingbirds in the northern California site had probabilities of apparent annual survival of 0.90 ± 0.03 (CI: 0.81–0.95) for residents and 0.17 ± 0.05 (CI: 0.09–0.30) for migrants.

### (d) Probability of persistence, probability of entry and intensity of availability

Probability of monthly persistence was modelled as a function of site, species, estimated individual time-since-arrival (TSA), the quadratically transformed monthly interval in the primary period (quad), as well as the interaction between species and quad (electronic supplementary material, table S1). Because residents were assumed to have a probability of persistence of 1, Φ estimates of persistence probability through the primary period are derived only for migratory birds.

Anna’s hummingbirds had significantly higher probabilities of monthly persistence than Allen’s hummingbirds ($\Phi_{\mathrm{Ann}a^{\prime }s}^{B}=0.8\pm 0.2$; CI: 0.3–1.2). Persistence was also significantly predicted by quad ($\Phi_{\mathrm{quad}}^{B}=-0.020\pm 0.005$; CI: 0.03–0.01) as well as the interaction between quad and species ($\Phi_{\mathrm{Ann}a^{\prime }s\;:\;\mathrm{quad}}^{B}=-0.016\pm 0.006$; CI: −0.028 to −0.004). Neither site ($\Phi_{\mathrm{site}}^{B}=0.31\pm 0.17$; CI: −0.02 to 0.64) nor estimated TSA ($\Phi_{\mathrm{TSA}}^{B}=0.06\pm 0.04$; CI : −0.02  to  0.14) significantly predicted persistence.

**Figure 2.**
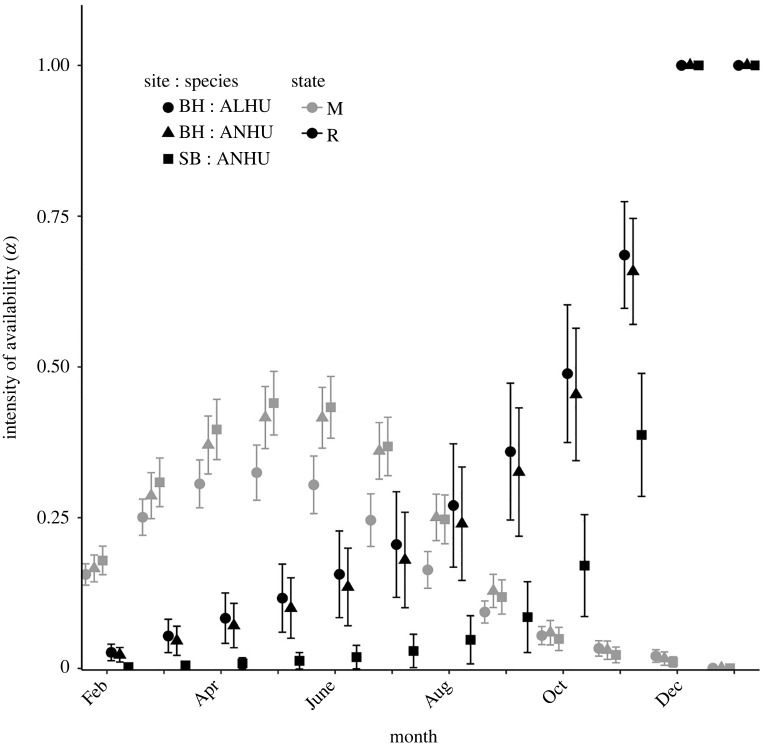
Intensity of availability of migratory (M) and resident (R) individuals in populations of Anna’s (ANHU; *C. anna*) and Allen’s (ALHU; *S. sasin*) hummingbirds for our northern (SB) and southern (BH) study sites in California, USA. Vertical error bars on point estimates are the upper and lower bounds of 95% confidence intervals.

Probability of monthly entry was estimated independently for every combination of site, species, migratory state and quadratically transformed monthly interval (electronic supplementary material, table S1). Probability-scale estimates of Φ and *β* were then used in the MSORD-SU model in MARK to derive estimates of the intensity of availability, which incorporates both the probabilities of entry and persistence to influence the periods when higher numbers of individuals are available for each state (figure 2). Therefore, intensity of availability is represented as a function of covariates of Φ and *β*, including month, species, site and migratory state.

### (e) Arrival, departure and residency times for migratory birds

**Figure 3.**
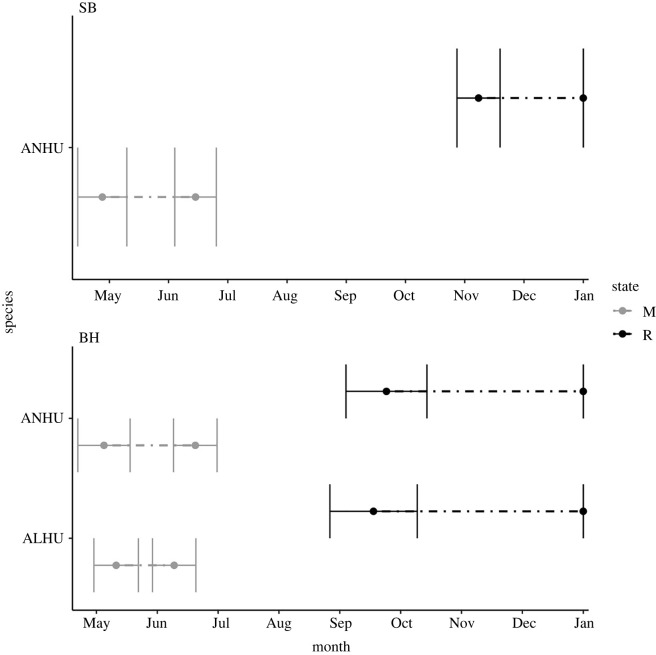
Estimated arrival and departure times of migratory (M) and resident (R) individuals in populations of Anna’s (ANHU; *C. anna*) and Allen’s (ALHU; *S. sasin*) hummingbirds for our northern (SB) and southern (BH) study sites in California, USA. Horizontal error bars on point estimates are the upper and lower bounds of 95% confidence intervals.

All residents were assumed to persist on the study area until the end of the annual period, although the entry times from the broader study region into the study area could occur in any month. Estimated arrival time for resident Allen’s hummingbirds in our southern population was 9.8 (CI: 9.3–10.3), corresponding to 25 October (9 October to 9 November), thus indicating that many individuals were entering the study area in all months even if assumed to be present in the broader region throughout the year. Resident Anna’s hummingbirds in the southern population arrived in period 9.8 (CI: 9.3–10.3), corresponding to 25 October (9 October to 9 November). In the northern population, resident Anna’s hummingbirds arrived in period 10.4 (CI: 10–10.8), corresponding to 12 November (1 November to 24 November) (figure 3).

Migratory Allen’s hummingbirds arrived in period 4.8 (CI: 4.4–5.2), corresponding to 25 May (12 May to 6 June) and departed in period 5.7 (CI: 5.3–6.1), corresponding to 21 June (9 June to 3 July). Migratory southern Anna’s hummingbirds arrived in period 4.4 (CI: 4.0–4.9), corresponding to 12 May (1 May to 28 May) and departed in period 5.9 (CI: 5.5–6.3), corresponding to 27 June (15 June to 9 July). Northern Anna’s hummingbirds arrived in period 4.6 (CI: 4.2–5.1), corresponding to 19 May (6 May to 3 June) and departed in period 6.4 (CI: 6.0–6.8), corresponding to 12 July (1 July to 25 July) (figure 3).

Residency times calculated using method 2, which is derived using only estimates of Φ, yielded estimates spanning the full primary period of 12 months for residents as there was a non-negligible probability that resident individuals would remain in the study area for the full annual period, thus violating one critical assumption of residency time derivation using method 2. We therefore report exclusively on *R*_1_ estimates, which are derived using estimates for both *β* and Φ.

Resident Allen’s hummingbirds were estimated to remain in our southern site for 3.2 mo ± 0.3 mo (CI: 2.71 mo to 3.73 mo), while migrants remained for 1.9 mo ± 0.1 mo (CI: 1.72 mo to 2.08 mo). Resident Anna’s hummingbirds were estimated to remain in our southern site for 3.2 mo ± 0.3 mo (CI: 2.71 mo to 3.74 mo), while migrants remained for 2.4 mo ± 0.1 mo (CI: 2.17 mo to 2.72 mo). Finally, resident Anna’s hummingbirds were estimated to remain in our northern site for 2.6 mo ± 0.2 mo (CI: 2.17 mo to 2.95 mo), while migrants remained for 2.7 mo ± 0.2 mo (CI: 2.41 mo to 3.07 mo).

### (f) Probability of detection and state classification

To model detection probability, *ρ*, we included terms for site, seasonal movement strategy and species (electronic supplementary material, table S1). Neither site ($\rho_{\mathrm{Site}}^{B}=-0.2\pm 0.2$; CI:−0.7  to 0.2) nor species ($\rho_{\mathrm{Ann}a^{\prime }s}^{B}=-0.2\pm 0.2$; CI :−0.6  to  0.2) significantly predicted detection probability. Seasonal movement strategy significantly influenced *ρ*, where resident birds were significantly less likely to be detected than migrants ($\rho_{R}^{B}=-0.8\pm 0.2$; CI : −1.1  to  −0.4). We nevertheless included site in our final model to account for differences in RFID detector placement relative to one another and to other resources in the northern and southern sites.

Detection probability of Allen’s hummingbirds in our southern California site was 0.74 ± 0.03 (CI: 0.67–0.80) if resident and 0.86 ± 0.02 (CI: 0.81–0.90) if migrant. Detection probability of Anna’s hummingbirds was 0.70 ± 0.04 (CI: 0.63–0.76) if resident and 0.83 ± 0.02 (CI: 0.78–0.87)) if migrant in our southern site and 0.64 ± 0.04 (CI: 0.55–0.73) if resident and 0.79 ± 0.03 (CI: 0.73–0.85) if migrant in our northern site.

Lastly, the MSORD-SU model includes a parameter for probability of being in each state, given that the state is unknown (*π*). We modelled *π* as a constant for resident birds (*π*^*B*^. = −1.9 ± 0.6; CI : −3.2 to −0.6). Birds caught before the winter months, which would, by our definition, classify them as residents, had a probability of 0.13 ± 0.07 (CI: 0.04–0.34) of being residents.

## 4. Discussion

We analysed remotely sensed RFID detection data comprising 330 Allen’s, 540 Anna’s and 177 black-chinned hummingbirds from two distinct populations in northern and southern California, USA (electronic supplementary material, figure S1). We leveraged our remotely sensed data and applied nascent statistical methods to quantify migratory behaviours in idiosyncratic hummingbird systems using single-study-site dynamics. With our single-site dynamics analyses, we exhibit the presence of migratory and resident strategies within species occupying the same area and that individuals can switch strategies throughout their lifetime. We also uncover starkly different estimates for apparent survival that may indicate a difference in propensity to permanently emigrate. Overall, our results highlight the complexity of movement strategies employed within a partial migrant system. The framework that we describe here could be used to rigorously quantify the population structure of any similar system wherein animals adopting different seasonal movement strategies coexist.

### (a) Hummingbird population structure and migratory behaviour

The migratory behaviours of Allen’s hummingbirds have changed over the past half century since the non-migratory subspecies (*S. s. sedentarius*) purportedly colonized mainland southern California [27]. Since the colonization of the mainland by this resident subspecies, non-migratory Allen’s hummingbirds expanded their range and it was postulated that the subspecies ranges did not overlap [27,45,46]. Our data demonstrate that this is false, as both migratory and resident Allen’s hummingbirds co-inhabit the same territory in our study area (figure 1). Moreover, individual Allen’s hummingbirds may adopt different seasonal movement strategies in consecutive years. This inter-individual variation in behaviour is increasingly recognized among diverse taxa, from fish [47], to insects [48], to ungulates [49], although the fitness trade-offs that govern divergent seasonal movement behaviours remain unclear. Adding to the complexity of our system, individual Allen’s hummingbirds at our southern California site exhibited migratory plasticity, where individuals adopted alternative seasonal movement strategy in year *t* and *t* + 1 with approximately equal probability of not changing strategy. Seasonal movements among the hummingbirds in our study areas are therefore more complex than previously presumed. Overall, we present evidence of facultatively controlled migratory behaviour in Allen’s hummingbirds (figure 1), while there is currently little conclusive genetic [46] or morphological [26,29] evidence of distinct subspecies. We therefore suggest that factors other than subspecies dynamics are primarily driving differences in seasonal movement patterns among populations of Allen’s hummingbirds.

We did not observe site-specific differences in proportions of migratory and resident Anna’s hummingbirds. Consistent with modern understanding of altitudinal [19], latitudinal [16], and partial migration in urban systems [50], we observed probabilities of adopting alternative movement strategies that were significantly higher than 0, supporting a facultatively controlled migratory behaviour. Our southern site, located in Beverley Hills, is a suburban habitat with high density of supplemental resource provisioning by humans, which can impact migratory ecology of some species [51]. Despite this, no significant difference in proportions of migrants and residents or probabilities of adopting alternative movement strategies in consecutive years was observed between sites. This suggests that habitats in both sites have seasonally limiting factors for hummingbird populations, though further study into habitat composition, sex and age class differences may be necessary to identify these factors. Owing to model limitations, we were not able to delineate residents that occupy the study area following post-breeding dispersal and individuals that occupy the site year-round by our definition of the resident strategy. This limitation may preclude precise site-specific estimation of proportions of truly stationary individuals. Therefore, further study is needed to draw inferences about the relative seasonal carrying capacity of these two habitats. Overall, we demonstrate that consideration for migratory plasticity in complex seasonal systems like ours is imperative for a complete understanding of population dynamics [20].

### (b) Probabilities of survival

Apparent survival estimates are negatively biased when rates of permanent emigration in a marked population are high [52,53]. Our survival estimates for resident birds that remain in the study area throughout the winter agree closely with published estimates of apparent Anna’s hummingbird survival after accounting for transients with the inclusion of a time-since-marking effect [23]. Apparent survival in the migrant class was significantly lower than the resident class, consistent with a meta-analysis of the fitness trade-offs between these two movement strategies in birds [16]. Overall, survival of migrants was lower than we expected based on the comparatively high probabilities of adopting this movement strategy and high proportions of migrants in the population overall. We posit that permanent emigration is therefore occurring at a much higher rate among the migrant class, thus negatively biasing apparent survival more so than appears to be the case for the resident class. Higher rates of permanent emigration in the migrant class could signify another seasonal movement strategy along a continuum with nomadism [54], which is driven by unpredictable resource availability. Apparent survival was also significantly lower in the suburban habitat relative to the comparatively rural site, which could also be due to a higher rate of permanent emigration. Urbanization is facilitated in species exhibiting migratory plasticity [50]; therefore, this migratory plasticity may be further adaptive for hummingbirds in urban environments, where resource availability is driven to the greatest extent by urban density and development, such that hummingbirds would permanently resituate when urban resources are developed, relocated or destroyed. Further study is still needed on the density-independent and density-dependent drivers of movement strategies.

### (c) Intensity of availability

The monthly intensity of availability (*α*) is expressed as the cumulative probability that individual *i* in month *j* arrived in any of the previous months and persisted on the study area [32]. Therefore, this derived parameter is a formulation of the arrival (*β*) and persistence (Φ) parameters. Our data were remotely sensed continuously throughout the year and probabilities of detection were high, as is generally the case in remotely sensed mark-detection data [55], and were higher than mark–recapture studies with hummingbirds [23]; therefore, hummingbirds were highly likely to visit at least one of the reader stations within the month if they were present in the population. Thus, we consider that our ability to detect the re-entry and persistence of previously tagged birds was high compared to recapture or resighting studies.

Estimates of *α* revealed annual patterns of availability consistent with our definitions of migratory and resident birds, where availability of migratory birds peaked between May and June and approached 0 in winter months. Conversely, resident availability increased continuously throughout the primary period and attained maximum availability in winter, as residents were expected to persist on the study area November through January.

One advantage of using remotely sensed tagging data for analyses with the MSORD-SU model is that there is minimized negative bias in the derived phenological parameters [32]. Since detection of previously tagged birds in the study area is high (*ρ* = 0.57 : 0.84) [56], low tagging effort is not expected to strongly influence arrival estimates. However, because our primary and secondary sampling periods are configured over a contiguous temporal range, mortality during secondary sampling periods is a source of negative bias in estimates of both Φ and *ρ* [32]. Nevertheless, using the MSORD-SU modelling framework for the analysis of banding data is subject to both forms of negative bias in phenological estimates [32] and we therefore suggest that remotely sensed RFID analyses using these methods provide nuanced and superior estimates than might otherwise be possible in alternative sampling regimes.

### (d) Arrival, departure and residency times

Our derived phenological estimates for resident and migrant hummingbirds yielded estimates of two clearly delineated groups of birds, although the residents in our study area may have included individuals that entered the study area during the post-breeding stage in addition to year-round residents. Migrants are likely present in the study area during breeding and then depart rapidly afterwards. Consistent with this interpretation of migrant and resident classes, some residents in our study area entered the population after the mean expected departure date of migrant birds. Given our modelling assumptions, residents may include birds adopting multiple possible seasonal movement strategies due to the highly plastic breeding phenology of Anna’s and Allen’s hummingbirds throughout California. Given the variability in breeding phenology of these hummingbirds, residency time, arrival and departure estimates may be subject to both positive and negative biases due to incorrect class assignment.

For migrants, probability of arriving is highest in the first monthly interval of the annual period, corresponding to the interval between February and March, after which time *β*^*M*^ declines steadily to approach 0 in winter months. We acknowledge that a migratory individual that arrived in our study sites in winter could cause underestimated residency and overestimated arrival times in the resident class but due to modelling limitations with the MSORD-SU framework, we were unable to assign different annual periods to different species in our system. We therefore selected winter months that most closely aligned among the three study species in our system. Derived estimates suggest a mean arrival date for migrant Allen’s hummingbirds as late as 25 May. Northbound migrating Allen’s hummingbirds reportedly pass through southern California as early as mid-January through mid-March [26], notably much earlier than our migrant class of Allen’s hummingbirds (figure 3). Still, the departure dates of our migrant Allen’s hummingbirds around 21 June suggest that this group corresponds to the conventional migrant group with wintering grounds in Mexico [26]. Allen’s hummingbirds’ migratory phenology is regulated in part by seasonally variable weather and resource availability [26] and therefore estimate precision may be improved in future analyses by including more than 3 years of data.

As a partial migrant species, Anna’s hummingbirds exhibit diverse migratory behaviours among different populations [24]. Our Anna’s hummingbird population in southern California exhibited similar arrival and departure timing to Allen’s hummingbirds (figure 3). Migratory Anna’s hummingbirds show highly similar arrival, departure and residency time estimates between the northern and the southern population. These individuals may undertake altitudinal, latitudinal, or southwesterly migration [24], though the departure time coincides with posited departures from southern California to Arizona [57]. Conversely, the mean winter resident arrival times from October to late November coincide with decreases in abundance in Arizona, as temperatures decline [24].

Expected residency times for resident birds on these sites were generally higher than for the migrant groups, although this pattern was not observed among Anna’s hummingbirds in our northern site. Our southern site is more suburban and developed than the northern site; ephemeral food resources in the north may limit bird abundance more strongly than in the suburban southern site during winter. Additionally, the southern site may have both more moderate climate and greater resource availability due to greater frequency of resource provisioning by humans. Further study is needed to determine the drivers of seasonal limitations to the relative carrying capacity of these habitats for stationary individuals.

### (e) RFID tag data for capture–recapture analyses

Overall, estimates of detection probability in this study were comparable to other studies with monthly estimates for among hummingbirds using RFID data [36]. Estimates were higher than those of studies using only banding data for probability of monthly detection [58] or annual detection [23], as is expected for effective remotely sensed data [55,59]. There are numerous advantages to applying RFID detection data to mark–recapture analysis beyond improved probability of detection. RFID marking has the potential for precise estimation of arrival, departure, and residency times [60], provided that animals were tagged before arriving on the site, or by including time-since-marking and age effects. Furthermore, data acquisition with RFID tags and readers requires comparatively minimal human labour [61]. Although several studies have not found short-term impacts of modern lightweight RFID tagging on survival [23] or flight activity [62], further study is needed to determine the effects of drag on energy budgets of free-ranging hummingbirds [62]. Moreover, subcutaneous implantation of RFID tags has inherent risk for birds’ health; detailed examination of the effects of the procedure on behaviour are warranted.

## 5. Conclusion

The relevance of our study to ecologists and ornithologists is twofold. Firstly, we present a framework for quantifying proportions of individuals within a population undertaking one of theoretically any number of definably distinct migratory behaviours using remotely sensed RFID data. With these methods, we present evidence that disambiguates the migratory habits of Allen’s and Anna’s hummingbirds using single-study-site dynamics. Importantly, both Allen’s and Anna’s hummingbirds using the same breeding location may adopt either resident and migratory strategies. This supports the existence of multiple migratory behaviours and refutes the existence of distinct subspecies among Allen’s hummingbirds in our study area. We observed species-specific probabilities of adopting alternative movement strategies in consecutive years, lending support to the existence of facultatively controlled migratory behaviour in both Allen’s and Anna’s hummingbirds. Finally, we observed distinct apparent survival estimates among the seasonal movement strategies, where wintering residents had significantly higher probabilities of apparent survival. We attribute this in part to a putatively higher proportion of permanent emigrants in the migrant class. Future studies may aim to establish a stronger understanding of the factors that drive migratory plasticity, both among hummingbirds in our study regions, but also for migratory species more generally. The importance of understanding the idiosyncratic seasonal movements of birds is paramount for ecological conservation.

## Data Availability

Electronic supplementary material, data and R code to analyse processed data are provided on the Dryad Digital Repository: https://doi.org/10.5061/dryad.n8pk0p2xw [63].
